# Supplementary material for: Impact of double-bolus tracking to individualize scan timing of the portal venous phase in preoperative computed tomography colonography angiography for right-sided colon cancer
Source: PLoS One. 2025 Mar 25;20(3):e0320630. doi: 10.1371/journal.pone.0320630 (PMC11936195; doi:10.1371/journal.pone.0320630)
Supplement: S1 Table — (DOCX) [file pone.0320630.s001.docx]

**S1 Table. CT values and contrast-to-noise ratios for accessory right colic vein.**

|  | DBT group | Fixed-delay group | *p*-value |
| --- | --- | --- | --- |
| CT value (HU) ^※^ |  |  |  |
| ARCV | 144.2 ± 9.3 | 135.1 ± 6.7 | 0.447 |
| Contrast-to-noise ratio^※^ |  |  |  |
| ARCV | 13.6 ± 1.5 | 11.5 ± 1.4 | 0.323 |

※ Data are presented as mean ± standard error.

CT, computed tomography; HU, Hounsfield unit; DBT, double-bolus tracking; ARCV, accessory right colic vein
